# Antidepressant and Anxiolytic-Like Effects of the Stem Bark Extract of *Fraxinus rhynchophylla* Hance and Its Components in a Mouse Model of Depressive-Like Disorder Induced by Reserpine Administration

**DOI:** 10.3389/fnbeh.2021.650833

**Published:** 2021-06-17

**Authors:** Yu Ri Kim, Bo-Kyung Park, Chang-Seob Seo, No Soo Kim, Mi Young Lee

**Affiliations:** ^1^Herbal Medicine Research Division, Korea Institute of Oriental Medicine, Daejeon, South Korea; ^2^Clinical Medicine Division, Korea Institute of Oriental Medicine, Daejeon, South Korea

**Keywords:** reserpine, depressive-like disorder, *F. rhynchophylla* Hance, antidepressant, anxiolytic, neuroinflammation, neuroprotection

## Abstract

There is an urgent need to find antidepressants that can be administered for long periods without inducing severe side effects to replace conventional antidepressants that control monoamine levels, such as tricyclic antidepressants (TCAs), monoamine oxidase inhibitors (MAOIs), and selective serotonin reuptake inhibitors (SSRI). We sought to determine the antidepressant effects of *Fraxinus rhynchophylla* Hance (*F. rhynchophylla* Hance, FX) and its components on a reserpine-induced mouse model. One hour after oral administration of FX (30, 50, and 100 mg/kg), esculin (50 mg/kg), esculetin (50 mg/kg), fraxin (50 mg/kg), and fluoxetine (20 mg/kg), reserpine was delivered intraperitoneally to mice. Behavioral experiments were conducted to measure anxiety and depressive-like behaviors after 10 days of administration. FX and its components increased the number of entries into the center of an open field as well as distance traveled within it and decreased immobility duration in the forced swim and tail suspension tests. Reserpine-induced increases in plasma corticosterone concentrations were attenuated by the administration of FX and its components, which were also found to decrease the reserpine-induced enhancement of mRNA levels of *interleukin (IL)-12 p40, IL-6*, and *tumor necrosis factor (TNF)-*α, pro-inflammatory cytokines. Finally, the diminished expressions of hippocampal phosphorylated cAMP response element-binding protein (pCREB) and brain-derived neurotrophic factor (BDNF) by reserpine were increased by FX and its components. Our results suggest that FX and its components regulate anxiety and depressive-like behaviors through stress hormones, immune regulation, and the activation of neuroprotective mechanisms, further supporting the potential of FX and its components as antidepressants.

## Introduction

Despite having a global prevalence of 350 million people and a long time-course, depression is not being treated effectively due to stigma and lack of effective therapeutic modalities (Smith, 2014). Depression is a complex mood disorder that manifests as despair and helplessness, all of which can further negatively impact health and mental by changes in appetite, abnormality in social behavior, insomnia, fatigue, frequent headaches, etc., if depressive mood conditions are prolonged (World Health Organization, 2009; Otte et al., 2016; Ikram and Haleem, 2017). The difficulty in treating depression is partly attributable to its varied etiology: genetic factors, environmental factors such as endocrine abnormalities, stress, sex differences, disease (stroke, cancer) (Laoutidis and Mathiak, 2013; Robinson and Jorge, 2016; Hammen, 2018), and especially biochemical factors such as neurotransmitter (norepinephrine, serotonin, GABA, etc.) and neural hormone (thyroid, growth, hypothalamus-pituitary-adrenal cortex axis) abnormalities can all increase the risk of the onset of depression (Saveanu and Nemeroff, 2012; Dell’osso et al., 2016). Antidepressants currently treating depression, tricyclic antidepressants (TCAs), monoamine oxidase inhibitors (MAOIs), and selective serotonin reuptake inhibitors (SSRI), which control existing monoamine levels, have side effects such as constipation, decreased vision, high blood pressure, cognitive impairment, and anticholinergic effects (Donoghue and Tylee, 1996; Feighner, 1999; Tarleton et al., 2016). Furthermore, while such early antidepressants are fast-acting and inexpensive, clinical studies have associated their use with low recovery rates, only 22–40% of patients with depression (Anthes, 2014).

*Fraxinus rhynchophylla* Hance (*F. rhynchophylla* Hance, FX) is a traditional Chinese medicine mainly found in China and Korea. FX contain coumarin-based components such as esculin, esculetin, and fraxin, and the stem bark obtained from FX—also known as fraxini cortex—has demonstrated efficacy in the treatment of diseases such as acute conjunctivitis, diabetes mellitus, diuretic, analgesic, astringent, and acute liver injury (Guo et al., 2017; Seo et al., 2019). Coumarin-based components isolated from *Fraxinus rhynchophylla* have been reported to have anti-inflammatory effects such as inhibiting tumor necrosis factor (TNF)-α release by peritoneal macrophages induced by LPS and attenuating the production of inflammatory mediators in BV2 microglia (Niu et al., 2012; Song et al., 2014). Among them, esculetin attenuates LPS-induced anxiety and depressive-like behavior and plays a role in inhibiting corticosterone and pro-inflammatory cytokines interleukin (IL)-1β, -6, and TNF-α (Sulakhiya et al., 2016). FX and its components improve depressive-like behavior and anti-inflammatory effects have been studied in several studies as above.

Reserpine was used as a first-line treatment for hypertension, but after chronic use, severe side effects leading to depression were observed (Guo et al., 2015). Studies have shown that this side effect is caused by the depletion of monoamines such as serotonin, dopamine, and norepinephrine (Gao et al., 2016). This evidence has been applied to animal models showing depressive symptoms, and reserpine-administered animal models are being used to study the pathological symptoms of depression (Uriguen et al., 2008). Reserpine administration may affect neuroinflammation by damaging rodent nerve tissues and releasing proinflammatory cytokines such as IL-1β, IL-12, IL-6, TNF-α, and IFN-γ from the hippocampus, liver, and serum (Huang et al., 2004; Zhou et al., 2014; Park et al., 2020). In addition, brain-derived neurotrophic factor (BDNF)-Tropomyosin receptor kinase B (TrkB) signaling participates in emotional, learning, and memory regulation in the hippocampus and activates neurotrophic pathways. Reserpine administration reduces BDNF expression and induces phosphorylation of BDNF binding receptor TrkB, leading to brain dysfunction, which plays an important role in depression (Zong et al., 2019). These neuroinflammatory reactions and neurotrophic factors have been proposed as causes of depression and are being studied as therapeutic targets.

Although our previous studies have identified the FX extract to be effective in attenuating stress-induced depression, depression is not considered a single disease; further research of the effect of FX in different models of depression was warranted. The present study specifically considers a reserpine-induced animal model to examine the applicability of prior findings to depression by environmental causes such as stress and dearth of neural monoamines. Hence, the present study first quantified the components by subjecting FX to high-performance liquid chromatography (HPLC) and then measured depression and anxiety-like behaviors and blood stress hormone levels. In addition, we examined the neuroinflammatory and neuroprotective effects of FX and its components on the hippocampal levels of the following elements: IL-12 p40, IL-6, TNF-α, and cAMP response element-binding protein (CREB)/BDNF. Therefore, our findings are expected to help establish FX and its components as natural products that improve depressive and anxiety-like symptoms, as well as mental disorders benefited by anti-inflammation and neuroprotection.

## Materials and Methods

### Chemicals and Antibodies

For HPLC analysis, esculin (EC, purity 98%) and esculetin (ECT, purity 98%) were purchased from Sigma-Aldrich (St. Louis, MO, United States). Fraxin (FR, purity 98.9%) and formic acid (analytical reagent grade) were purchased from Merck KGaA (Darmstadt, Germany). For animal experiments, reserpine (purity 98%), esculin (purity 98%), esculetin (purity 98%), and fluoxetine (FXT, purity 98% in thin layer chromatography) were supplied by Sigma-Aldrich. Fraxin (purity 98%) was supplied by InterPharm Corporation (Koyang-si, Gyeonggi-do, South Korea). For anesthesia, tiletamine/zolazepam was supplied by Virbac (Zoletil 50; Cedex, France). For western blot analysis, actin antibody was supplied by Sigma- Aldrich. BDNF antibody was supplied by Abcam plc. (Cambridge, United Kingdom). CREB antibody and phosphorylated CREB (pCREB) antibody were supplied by Cell Signaling Technology (Danvers, MA, United STates). For immunofluorescence analysis, BDNF antibody was supplied by Abcam plc, pCREB antibody by Cell Signaling Technology, and NeuN antibody by Merck KGaA.

### Preparation of the FX Extract

The stem bark obtained from FX, the origin of Gyeongsangbukdo of Korea, was supplied by Omniherb Co., Ltd. (Susung-gu, Deagu, South Korea). One kg of FX stem bark was submitted to reflux extraction for 3 h with 10 L of 70% ethanol solvent. After filtering the primary strainer, it was concentrated by secondary cotton filtration to freeze-dry the extract and prepare a powder. Approximately 171.86 g of ethanol extract was obtained, and the yield of this extract was 11.46%. The extract was stored at −80°C.

### HPLC Reagents, and the Analysis of the FX Sample

The phytochemical analysis of FX was performed using a Shimadzu Prominence LC–20A system (Kyoto, Japan) equipped with a photodiode array (PDA) detector. LC solution software (Version 1.24, SP1, Kyoto, Japan) was employed for the acquisition, processing, and conversion of chromatographic data. A Waters SunFire C_18_ column (250 × 4.6 mm, 5 μm, Milford, MA, United States) maintained at 40°C was used to separate the three marker components in the FX sample. The mobile phases consisted of 0.1% aqueous formic acid and 0.1% (v/v) formic acid in acetonitrile. The gradient elution of the mobile phase was as follows: 5–60% B for 0–40 min, 60% B for 40–45 min, and 60–5% B for 45–50 min. The flow-rate and injection volume were 1.0 mL/min and 10 μL, respectively.

### Animals

Seven-week-old male c57BL/6 mice were purchased from DBK Co., Ltd. (Eumseong-gun, Chungcheongbuk-do, South Korea). The mice were housed in specific-pathogen-free (SPF) conditions at a constant temperature and underwent a week-long adaptation period in 12/12 h light/dark cycles. The mice were fed a commercial diet (Cargill, Incorporated., Pyengtaek-si, Gyonggi-go, South Korea) and allowed tap water *ad libitum* throughout the study. All experiments were approved by the Committee on Animal Care of KIOM (17-104) and Use Committee in accordance with the National Institutes of Health Guidelines (NIH).

### Treatments and Groups

**FIGURE 1:**
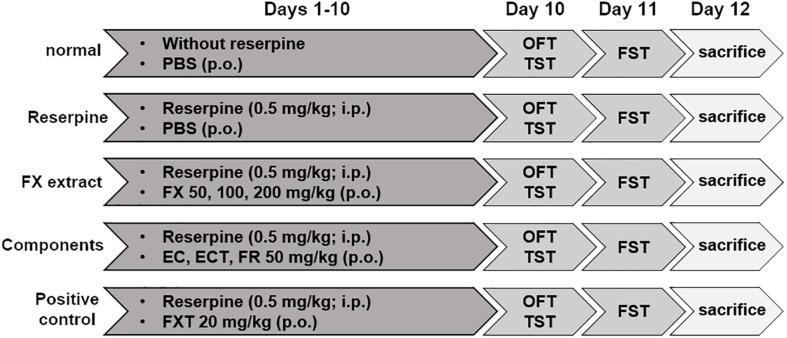
Schematic diagram showing the experimental design. Drugs were orally and intraperitoneally administered from days 1 to 10. The OFT, TST, and pre-FST were performed on day 10. The FST was conducted on day 11. All mice were sacrificed on day 12.

Before the experiment began, reserpine, FX, EC, ECT, FR, and FXT were dissolved in PBS, and dispensed into 1.5 ml tubes with the amount to be used per day. The dispensed drugs were stored at −20°C, and used one by one on the day of administration. Reserpine was intraperitoneally administered to the mice at a concentration of 0.5 mg/kg (in PBS containing 0.1% dimethyl sulfoxide and 0.3% Tween-80) at a 100 μl dose to induce anxiety and depressive-like behaviors. They were randomly divided into the following nine groups: normal (non-reserpine + PBS), reserpine (reserpine + PBS), FX 30 (reserpine + FX 30 mg/kg), FX 50 (reserpine + FX 50 mg/kg), FX 100 (reserpine + FX 100 mg/kg), EC (reserpine + esculin 50 mg/kg), ECT (reserpine + esculetin 50 mg/kg), FR (reserpine + fraxin 50 mg/kg), and FXT (reserpine + fluoxetine 20 mg/kg). Mice received PBS, FX, EC, ECT, FR, and FXT orally at a 100 μl dose, according to their groups, once a day for a total of 10 days. A schematic of the experimental schedules is shown in Figure 1 (Park et al., 2018; Yu et al., 2019).

### Body Weighing, and Anxiety and Depressive-Like Behavior Tests

Body weight was measured before the experiment (basal) and 5 and 10 days after the beginning of the experiment. The measured increase in body weight was calculated as a percentage of the basal measurement. Anxiety and depressive-like behaviors were measured with open field (OFT), forced swim (FST), and tail suspension tests (TST). OFT measures the activity of mice exposed to a new environment to identify anxiety symptoms. The mice were placed in a white acrylic box (30 × 30 × 40 cm) and recorded for 10 min with EthovisionXT9 (Noldus Information Technology, Wageningen, The Netherlands). The degree of anxiety was measured by analyzing the distance traveled and the number of times the center of the arbitrarily-set acrylic box’s central zone (10 × 10 cm) was crossed. FST and TST gauged despair symptoms. During FST, mice were placed in a transparent cylindrical cylinder filled with water at 25°C (H: 45 cm, D: 20 cm). Mice were exposed to the water tank for 15 min the day before the experiment to induce despair and lethargy (Marks et al., 2009), and improvement in behaviors such as induced despair produced by FX and its components confirmed with this experiment the next day. TST measures immobile time while the mouse is suspended with a tape attached to the tail from the top of a 50 cm high white acrylic box. In FST and TST experimental trials, the total recording time was 6 min; the immobility time during the last 4 min was measured using a video-tracking software (SMART 3.0; Panlab S.I., Barcelona, Spain).

### Enzyme-Linked Immunosorbent Assay (ELISA)

On day 12, the mice were anesthetized with tiletamine/zolazepam (25 mg/kg), and blood was collected from the heart. Blood was centrifuged at 3,000 rpm at 4°C for 10 min. The separated supernatant plasma was transferred to another tube and stored at −70°C. Corticosterone concentration in plasma was determined with the corticosterone ELISA kit (Cayman chemical company, Ann Arbor, MI, United States). All experiments using this kit were performed according to the manufacturer’s protocols. Corticosterone concentration was identified with a VersaMax microplate reader (Molecular Devices, Sunnyvale, CA, United States), and the absorbance was measured at the appropriate optical density using SoftMax pro 6.2.2 (Molecular Devices).

### Real-Time Polymerase Chain Reaction (qPCR)

In the hippocampus, RNA was isolated with easy-BLUE^TM^ reagent (iNtRON Biotechnology, Seongnam-si, Gyeonggi-do, South Korea), and cDNA synthesized in equal amounts with PrimeScript RT reagent kit (TaKaRa, Shiga, Japan). The base sequences of the primers used in real-time PCR are shown in Table 1. The cDNA was loaded onto MicroAmp Fast 96-well reaction plates (Applied Biosystems, CA, United States) with each primer and SYBR Green PCR Master Mix (Applied Biosystems). mRNA was measured with Quantstudio 6 Flex (Applied Biosystems).

**TABLE 1:** Real-time PCR primer sequences.

Gene	Sequence	
	Forward	Reverse
Mouse IL-12 p40	5′-AGACATGGAGTCATAG GCTCTG-3′	5′-CCATTTTCCTTCTTGTG GAGCA-3′
Mouse IL-6	5′-GAGGATACCACTCCCAA CAGACC-3′	5′-AAGTGCATCATCGTTG TTCATACA-3′
Mouse TNF-α	5′-AGACCCTCACACTCAGATC ATCTTC-3′	5′-CCACTTGGTGGTTT GCTACGA-3′
Mouse GAPDH	5′-AAGGTGGTGAAGC AGGCAT-3′	5′-GGTCCAGGGTTTCTT ACTCCT-3′

**IL, interleukin; TNF, tumor necrosis factor; GAPDH, glyceraldehyde-3-phosphate dehydrogenase.**

### Western Blotting

The hippocampus was homogenized in 500 μl RIPA buffer (Thermo Fisher Scientific, Waltham, MA, United States) with Pro-Prep^TM^ (iNtRON Biotechnology) and equalized to the same amount (20 μg) of protein. The equalized samples were separated with 4–20% Mini-PROTEAN TGX Precast Protein Gels (Bio-Rad Laboratories, Inc., Hercules, CA, United States), and separated proteins were transferred to a PVDF (Amersham Biosciences, Piscataway, NJ, United States). Membranes were blocked in 5% skim milk (Bio-Rad Laboratories, Inc.) solution for 1 h at room temperature and incubated with primary antibody overnight at 4°C: actin (Dilution ratio 1:2,000), BDNF (Dilution ratio 1:1,000), CREB (Dilution ratio 1:1,000), and pCREB antibodies (Dilution ratio 1:1,000). Membranes were subsequently incubated with appropriate secondary mouse and rabbit antibodies (Cell Signaling Technology) for 1 h at room temperature. Actin was used as a loading control for all experiments. The density of the protein band was quantified using an ImageQuant LAS 4000 mini (Fujifilm, Tokyo, Japan).

### Immunofluorescence

The whole brain was fixed in 4% paraformaldehyde solution (BIOSESANG, Seongnam, Gyeonggido, South Korea) and dehydrated in 30% sucrose (Samchun chemicals, Gangnam-gu, Seoul, South Korea) solution. The dehydrated brain was modeled with OCT compound (Leica Biosystems, Wetzlar, Germany) and stored at −70°C. Modeled brains were sectioned into 30 μm sections with a cryostat (Leica Biosystems) at −20°C and attached to glass slides (Paul Marienfeld GmbH & Co., Lauda-Königshofen, Germany). Brain sections were post-fixed with 4% paraformaldehyde solution for 15 min and blocked for 1 h in a blocking buffer (1 × PBS/5% normal goat serum/0.3% Triton X-100). BDNF, pCREB, and NeuN antibodies were diluted 1:500 in antibody dilution buffer (1 × PBS/1% BSA/0.3% Triton X-100) and incubated overnight at 4°C. FITC and Texas red-conjugated secondary antibodies (Invitrogen by life technologies, MA, United States) were incubated for 2 h at room temperature, and longitudinal nuclei were stained with VECTASHIELD^®^ Antifade Mounting Medium with DAPI (Vector Laboratories, Inc. CA, United States). Expressions were analyzed in the dentate gyrus of the hippocampus at 20 × (pCREB) and 40 × (BDNF) magnification. Imaging and IOD measurements were performed using a fluorescence microscope (Nikon Instruments Inc., Tokyo, Japan) and the NIS-Elements program (Nikon Instruments Inc.).

### Data Analyses

All data are expressed as mean ± standard deviation (*SD*) and analyzed using GraphPad Prism 7 (GraphPad Software, Inc., La Jolla, CA, United States). Statistical analysis was performed using one-way and repeated one-way analyses of variance (ANOVA) with Tukey’s *post hoc* comparisons. *P*< 0.05 were considered to indicate statistical significance.

## Results

### HPLC Analysis of FX Sample

**FIGURE 2:**
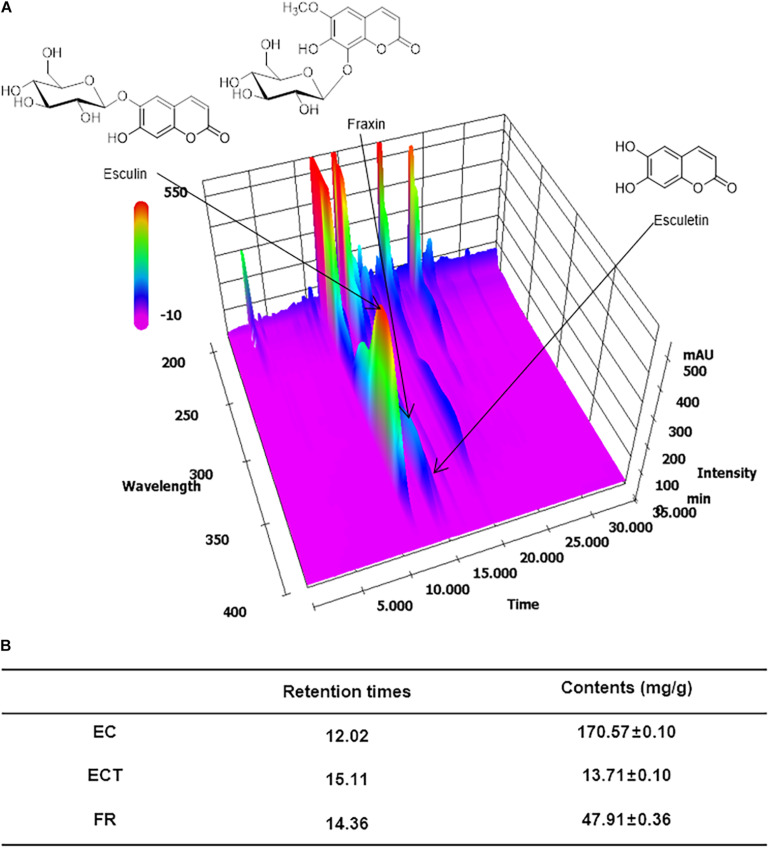
FX extract standardization *via* HPLC analysis**. (A)** Three-dimensional HPLC chromatogram of the FX sample. **(B)** Retention times and contents (mg/g) of EC, ECT, and FR.

The optimal HPLC analytical method was successfully applied for the quantification of three marker components in the FX sample. All analytes were separated for 20 min with a resolution of ≥ 4.7. Representing the HPLC chromatograms of the FX sample (Figure 2A), the retention times of esculin, esculetin, and fraxin were 12.02, 15.11, and 14.36 (Figure 2B), respectively. The coefficient of determination (*r*^2^) of the calibration curve of all analytes was 1.0000, indicating that the calibration curve shows excellent linearity. The regression equation, limit of detection (LOD), and quantitation (LOQ) values for the three marker components are presented in Table 2. Quantification of these analytes was performed at 335 nm for esculin, 340 nm for fraxin, and 345 nm for esculetin. The amounts of the three marker components (esculin, esculetin, and fraxin) in lyophilized FX sample were detected to be 170.57 ± 0.10, 13.72 ± 0.10, and 47.91 ± 0.36 mg/g, respectively.

**TABLE 2:** Linear range, regression equations, *r*^2^, LODs, and LOQs of the eight bioactive compounds.

Compound	Linear range (μg/mL)	Regression equation^*a*^	*r*^2^	LOD^*b*^ (μg/mL)	LOQ^*c*^ (μg/mL)
Esculin	0.78–50.00	y = 21843.50x + 2894.96	1.0000	0.10	0.31
Esculetin	0.78–50.00	y = 36254.96x + 3516.57	1.0000	0.12	0.37
Fraxin	1.56–100.00	y = 18621.50x + 5313.38	1.0000	0.22	0.67

**^*a*^y: peak area (mAU) of compounds; x: concentration (μg/mL) of compounds. ^*b*^LOD = 3.3σ × S. ^*c*^LOQ = 10σ × S. σ is the standard deviation of the y-intercept, and S is the slope of the calibration curve.**

### Effect of FX Extract and Its Components on Anxiety and Depressive-Like Behaviors

**FIGURE 3:**
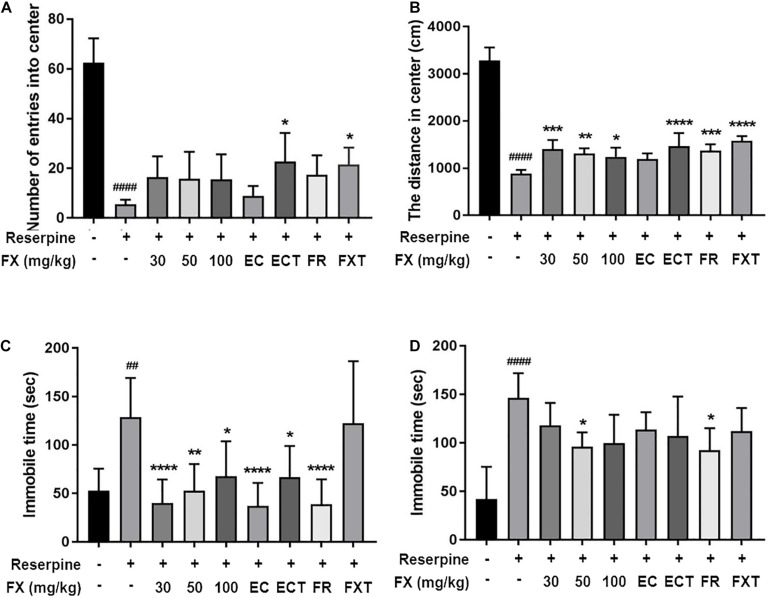
Effect of FX extract and its components on performance in the OFT, TST, and FST. Mice were subjected to the **(A,B)** OFT (*n* = 6), **(C)** TST (*n* = 6), and **(D)** FST (*n* = 8). FX extract, EC, ECT, and FR significantly improved reserpine-induced depressive behaviors. ^##^*P* < 0.01 and ^####^*P* < 0.0001 vs. normal group; *P < 0.05, **P < 0.01, ***P < 0.001, and ****P < 0.0001 vs. reserpine group.

We performed anxiety and depressive-like behavioral tests at 10 days following reserpine (i.p.), FX, components, and FXT (orally) administrations. FX treatment tended to increase the number of entries into the center [*F*(8, 45) = 23.84, *p* < 0.0001]; particularly, ECT and FXT treatment significantly increased the number of entries into the center (Reserpine: 5.571 ± 1.81; ECT: 22.67 ± 11.57, *p* < 0.01; and FXT: 21.5 ± 6.892, *p* = 0.033; Figure 3A). The distance traveled in the center [*F*(8, 45) = 88.73, *p* < 0.0001] significantly increased in the FX, ECT, FR, FXT-treated groups (Reserpine: 889.8 ± 73.59; FX 30: 1,405 ± 193.9, *p* = 0.0003; FX 50: 1,313 ± 108.7, *p* = 0.0048; FX 100: 1,241 ± 192.5, *p* = 0.0339; ECT: 1,468 ± 276.2, *p* < 0.0001; FR: 1,377 ± 127.2, *p* < 0.0001; and FXT: 1,579 ± 101, *p* < 0.0001; Figure 3B). Immobility time recorded during FST [*F*(8, 63) = 8.223, *p* < 0.0001] was significantly reduced in the FX, EC, ECT, and FR-treated groups (Reserpine:128.9 ± 40.4; FX 30: 40.32 ± 24.11, *p* < 0.0001; FX 50: 52.7 ± 27.68, *p* = 0.002; FX 100: 67.71 ± 36.27, *p* = 0.0271; EC: 37.04 ± 23.94, *p* < 0.0001; ECT: 66.84 ± 32.33, *p* = 0.0162; and FR: 38.74 ± 25.84, *p* < 0.0001; Figure 3C), and that recorded during TST [*F*(8, 45) = 6.536, *p* < 0.0001] was significantly reduced in the FX 50 and FR-treated groups (Reserpine: 146.6 ± 25.32; FX 50: 96.19 ± 14.64, *p* = 0.049; and FR: 92.55 ± 22.63, *p* = 0.0226; Figure 3D). These results suggest that treatment with FX extract and its components affects anxiety and depressive-like behaviors.

### Effect of FX Extract and Its Components on Body Weight and Stress-Related Hormones in Plasma

**FIGURE 4:**
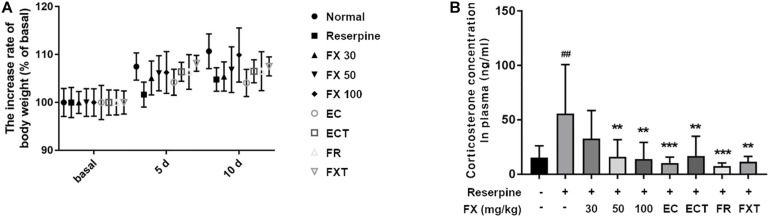
Effect of FX extract and its components on body weight and plasma corticosterone concentration. The **(A)** body weight (*n* = 8) and **(B)** plasma corticosterone concentrations (*n* = 8) of the mice were measured. FX extract tended to increase body weight. FX extract, EC, ECT, and FR significantly decreased corticosterone concentration in the plasma. = 8. ^##^*P* < 0.01 vs. normal group; **P < 0.01 and ****P < 0.0001 vs. reserpine group.

There were no significant differences between groups in the body weight measured at 5 and 10 days. However, the body weight measured at 10 days tended to increase in the FX 100-treated group relative to the reserpine group (Figure 4A). Because reserpine administration increases the concentration of the stress hormone plasma corticosteroid by 3–5 times (Lengvari and Halasz, 1972), the effect of FX extract and its components on the change in plasma corticosterone concentration was confirmed. Corticosterone, a stress-related hormone, was significantly decreased in the plasma of the mice in the FX 50, 100, EC, ECT, FR, and FXT-treated groups (Reserpine: 55.81 ± 44.98; FX 50: 15.95 ± 15.92, *p* = 0.0062; FX 100: 14.01 ± 15.35, *p* = 0.0033; EC: 10.18 ± 5.68, *p* < 0.0009; ECT: 16.81 ± 18.16, *p* = 0.008; FR: 7.457 ± 2.92, *p* = 0.0002; and FXT: 11.51 ± 4.88, *p* = 0.0015; Figure 4B). These results suggest that FX extract affects body weight, and FX extract and its component decrease the concentrations of plasma stress-related hormone levels on reserpine-induced mouse model.

### Effect of FX Extract and Its Components on Hippocampal Pro-inflammatory Cytokine mRNA Levels

**FIGURE 5:**
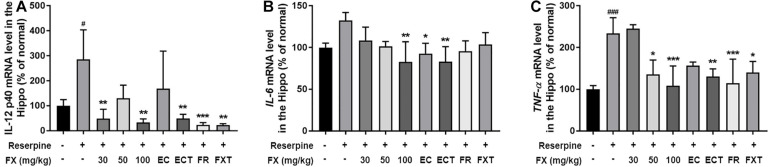
Effect of FX extract and its components on the expression of *IL-12 p40, IL-6*, and *TNF-*α mRNA levels. **(A)** *IL-12 p40*, **(B)** *IL-6*, and **(C)** *TNF-*α mRNA levels were measured in the hippocampus of the mice with real-time PCR (*n* = 3–4). FX extract, EC, ECT, and FR increased *pro-inflammatory cytokines* mRNA levels. ^#^*P* < 0.05 and ^###^*P* < 0.001 vs. normal group; *P < 0.05, **P < 0.01, and ***P < 0.001 vs. reserpine group.

Antidepressants used in the treatment of depression affect the concentration of pre-inflammatory cytokines and bring anti-inflammatory effects (Kopschina Feltes et al., 2017). The effects of FX extract and its components on the expression of pro-inflammatory cytokines were identified in the hippocampus. Reserpine administration significantly increased the mRNA levels of IL-12 p40 and TNF-α, and tended to increase the mRNA level of IL-6 (IL-12 p40: 319.4 ± 118.1, *p* = 0.0063; TNF-α: 233.6 ± 37.7, *p* = 0.0003; Figure 5). The FX 100-treated group attenuated all these increases (IL-12 p40: 33.57 ± 13.68, *p* = 0.0008; IL-6: 82.84 ± 24.05, *p* = 0.0037; TNF-α: 108.4 ± 47.57, *p* = 0.0006). The EC-treated group significantly restored IL-6 mRNA levels (92.71 ± 12.32, *p* = 0.0285), and the ECT-treated group significantly reduced IL-12 p40 (49.05 ± 16.96, p = 0.0014), IL-6 (83.01 ± 18, *p* = 0.0016), and TNF-α (130.6 ± 18.2, *p* = 0.0062) mRNA levels. The FR-treated group significantly reduced IL-12 p40 (23.54 ± 9.04, *p* = 0.0002) and TNF-α (114.5 ± 57.67, *p* = 0.0006) mRNA levels. Finally, the FXT-treated group showed significantly reduced IL-12 p40 (22.73 ± 5.222, *p* = 0.0005) and TNF-α (140.1 ± 26.58, *p* = 0.0162) mRNA levels. These results suggest that FX 100 mg/kg and its components regulate the expression of pro-inflammatory cytokines in the hippocampus of mice treated with reserpine.

### Effect of FX Extract and Its Components on CREB/BDNF Signaling in the Hippocampus

**FIGURE 6:**
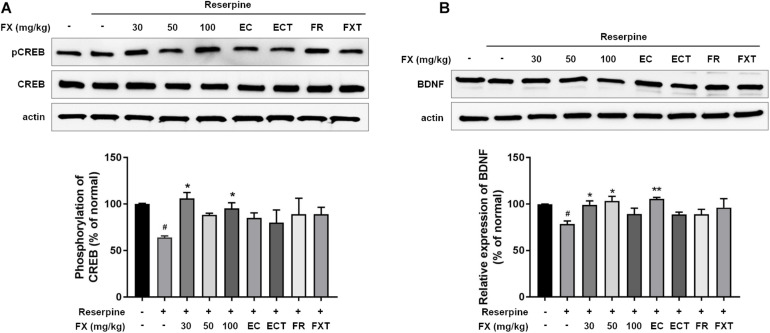
Effect of FX extract and its components on the expression of pCREB/CREB and BDNF. **(A)** pCREB/CREB and **(B)** BDNF levels were measured in the hippocampus. FX extract significantly increased pCREB/CREB levels (*n* = 3). FX extract EC significantly increased BDNF levels. ^#^*P* < 0.05 vs. normal group; *P < 0.05 and **P < 0.01 vs. reserpine group.

**FIGURE 7:**
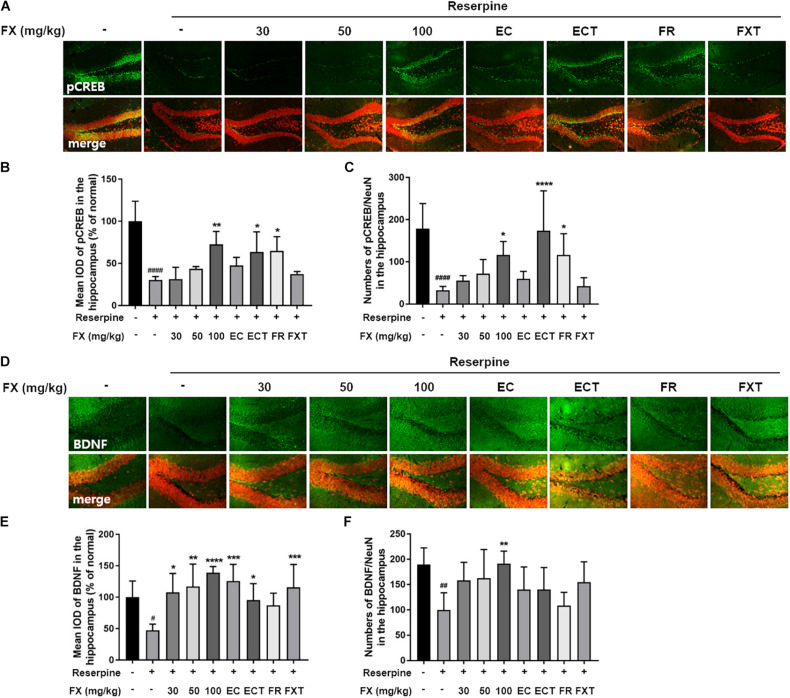
Effect of FX extract and its components on the expression of pCREB and BDNF in the hippocampal neuron. **(A–C)** pCREB and **(D–F)** BDNF level were measured in the dentate gyrus of the hippocampus with immunofluorescence (*n* = 5). FX extract, ECT, and FR significantly increased pCREB levels in the hippocampal neurons. FX extract, EC, and ECT significantly increased BDNF levels. In particular, FX extract significantly increased BDNF levels in the hippocampal neurons. ^#^*P* < 0.05, ^##^*P* < 0.01, and ^####^*P* < 0.0001 vs. normal group; *P < 0.05, **P < 0.01, ***P < 0.001, and ****P < 0.0001 vs. reserpine group.

We measured CREB/BDNF expression to determine the effects of FX extracts and its components on hippocampal neuroprotective mechanisms. Reserpine administration significantly reduced pCREB/CREB expression (64.17 ± 1.60, *p* = 0.0344; Figure 6). The decreased expression was restored with the administration of FX extract at doses of FX 30 and 100 mg/kg (FX 30: 106.1 ± 6.33, *p* = 0.011; FX 100: 97.31 ± 3.29, *p* = 0.0472). While BDNF expression decreased with reserpine (78.47 ± 3.35, *p* = 0.0314), FX 30, 50 mg/kg, and esculin significantly increased BDNF expression (FX 30: 99.23 ± 4.26, *p* = 0.0385; FX 50: 103.4 ± 4.99, *p* = 0.0129; EC: 105.6 ± 1.64, *p* = 0.0074). The location and cell type expressing pCREB or BDNF were measured using immunofluorescence. Changes in pCREB and BDNF expression were observed in the dentate gyrus of the hippocampus (Figures 7A,D). Reserpine administration decreased the pCREB expression (30.33 ± 4.09, *p* < 0.0001) and the number of pCREB positive neurons in the hippocampus (32.43 ± 9.27, *p* < 0.0001). However, FX 100, ECT, and FR-treated groups significantly restored the expression of pCREB (FX 100: 72.7 ± 15.41, *p* = 0.0024; ECT: 63.67 ± 23.84, *p* = 0.0395; and FR: 64.82 ± 16.95, *p* = 0.0279; Figure 7B) and the number of pCREB positive neurons (FX 100: 116.3 ± 32.05, *p* = 0.0231; ECT: 174.4 ± 94.02, *p* < 0.0001; and FR: 116.6 ± 50.16, *p* = 0.0359; Figure 7C). Reserpine administration also reduced BDNF expression (47.56 ± 9.65, *p* = 0.0251) and the number of BDNF positive neurons (99.86 ± 33.92, *p* = 0.0018). FX, EC, ECT, and FXT-treated groups significantly increased BDNF expressions (FX 30: 107.8 ± 30.16, *p* = 0.0136; FX 50: 117.2 ± 35.53, *p* = 0.002; FX 100: 139.3 ± 9.63, *p* < 0.0001; EC: 126 ± 26.46, *p* = 0.0001; ECT: 95.51 ± 26.31, *p* = 0.0472; and FXT: 115.7 ± 36.54, *p* = 0.0005; Figure 7E). In particular, FX 100-treated group significantly increased the number of hippocampal BDNF-positive neurons (191.67 ± 24.42, *p* = 0.0023; Figure 7F). These results suggest that the FX extract and its components may exert neuroprotection by increasing the expression of CREB and BDNF in the neurons of hippocampus dentate gyrus.

## Discussion

While our previous studies found that FX extract and its components ameliorate chronic stress-induced depression, the present investigation sought to determine whether FX extract and its components are effective when anxiety and depressive-like behaviors can be directly attributed to the biochemical downregulation of monoamine in addition to attendant environmental causes. Specifically, we explored the aforementioned question by administering FX and its components to a reserpine-induced mouse model. FX extract and its components were found to improve anxiety and depression-like behaviors—as measured through OFT, TST, and FST—and affected the expression of stress hormones that contribute to depressive and anxiety symptoms. Furthermore, FX extract and its components decreased pro-inflammatory cytokine mRNA levels elevated by reserpine administration and increased CREB/BDNF signaling. The present findings thus confirm that FX extract and its components have a beneficial effect on depressive states.

Reserpine-induced animal models of depression have been used in many other studies; reserpine has been recognized for over 50 years as an antihypertensive and psychotropic drug. However, its long-term use has been associated with side effects that cause depression by depleting neural concentrations of monoamines. The use of reserpine to emulate depression in animals is thus validated as a correlate of investigating depression in humans (Baumeister et al., 2003; Zhang et al., 2018). Therefore, in this study, we selected an animal model for depressive and anxiety like behavior caused by monoamine depletion by reserpine administration and tried to verify the antidepressant and anxiety relief efficacy of FX extract and its components.

Behavioral tests such as the OFT, TST, and FST are widely used to evaluate antidepressant and anxiolytic activity in animal models. OFT identifies anxiety-like behaviors by measuring search activity in new environments. At the same time, the FST and TST are thought to confirm depressive-like behaviors by stimulating the escape instinct of the mouse and confirming whether an active coping style has been retained. In these tests, increased activity in the center zone and reduced immobility indicates a drug’s antidepressant and anxiolytic effect (Chang et al., 2015; Wei et al., 2018). Reserpine administration improves anxiety and depressive-like behaviors shown by mice staying at the edge of the space in OFT and maintaining immobility in TST and FST (Chang et al., 2015). This induced symptom effectively relieves anxiety and depressive symptoms, such as increased crossing and decreased immobility by antidepressant-like PSAP and herbal medicines (Sousa et al., 2018; Park et al., 2020). This study found that reserpine-administered mice exhibited reduced central dwelling time and entry frequency in the OFT as well as increased immobility in the TST and FST. Our results, therefore, suggest that FX and its components attenuate reserpine-induced anxiety and despair, as measured in the OFT and FST/TST, respectively.

Corticosterone is a hormone secreted into the plasma from the adrenal cortex. CRH, secreted by stress, is regulated by the hypothalamus-pituitary-adrenal (HPA) axis. Corticosteroids (ACTH) are secreted by pituitary stimulation, increasing the concentrations of epinephrine and corticoids in the adrenal glands (Petrescu et al., 2018). Stress is thus considered a major cause of depressive symptoms. Stressed animals reportedly feature increased blood concentrations of ACTH (Yang et al., 2015). Reserpine administration has further been observed to increase the level of corticosterone in the blood. The increase in stress hormones due to monoamine reduction is reduced by psychotropic drugs (Mitra et al., 1977; Park et al., 2018). In addition, plasma corticosterone has been shown to increase in LPS induced despair-like mice, another depressive animal model, and be significantly reduced by various antidepressants (Tomaz et al., 2020). Our study further showed that treatment with FX and its components significantly reduced plasma corticosterone concentrations. These results are thought to implicate corticosterone activity and stress elevation in the biochemical effects of reserpine; however, this hypothesis requires further validation.

While the mechanism of action of FX on depressive-like behaviors in the treatment of depression remains unknown, antidepressants have been shown to affect monoamine activity as well as feature anti-inflammatory and neuroprotective effects (Kopschina Feltes et al., 2017); specifically, antidepressants reportedly affect the production of cytokines (Obuchowicz et al., 2014). Several studies have further found that the hippocampal concentrations of the pro-inflammatory cytokines IL-6, IL-1β, and TNF-α are elevated in mice with depression-like behavior (Goshen et al., 2008; Mohamed et al., 2013; Hsieh et al., 2014; Tao et al., 2016). In addition, IL-12 was significantly increased in the plasma of patients with major depressive disorder (Lee and Kim, 2006). Reserpine, which induces anxiety and depression, increased levels of proinflammatory cytokines, TNF-α, IL-6, IL-12, and IFN-γ, mRNA, which induce inflammation and regulate immune cells in serum (Li et al., 2014). In addition, hippocampal pro-inflammatory cytokines in depressive states due to chronic unpredictable mild stress were significantly down-regulated by the administration of FXT, an SSRI antidepressant (Fernandes and Gupta, 2019; Shen et al., 2019). Our study also increased the mRNA levels of IL-12 p40, IL-6, and TNF-α increased by administration of reserpine, thus confirming that FX and components reduced these inflammatory cytokines. These results suggest that the antidepressant effects of FX extract and its components may be involved in neuroinflammation, accompanied by the reduction of IL-12, IL-6, and TNF-α mRNA levels.

The hippocampus plays an important role in learning and memory and is known as a major regulator of stress and mood. However, while the role of hippocampal neurons in depressive and stress disorders has been extensively studied, the exact mechanism underlying their involvement remains unclear (Planchez et al., 2020). The reported influence of antidepressants on synaptic and dendritic remodeling, which underlies antidepressant effects, supports the hypothesis that an effective antidepressant treatment could address neural developmental mechanisms (Segi-Nishida, 2017; Duman et al., 2019). Furthermore, stress-depressed mouse models show that decreased hippocampal levels of BDNF, TrkB, PI3K, and CREB induce neurological damage (Nestler et al., 2002; Wu et al., 2017; Jiang et al., 2019). In addition, the reserpine administration model reduced the levels of BDNF and CREB mRNA in the hippocampus and, in particular, showed a biochemical change of BDNF. Antidepressant administration reduces BDNF and has been shown to affect nerve cell damage recovery (El-Marasy et al., 2021). Our results confirmed that FX extract increased BDNF expression and CREB activity, particularly in hippocampal neurons. These results suggest that the administration of FX extract and its components can modulate BDNF/CREB signaling in hippocampal neurons. However, further validation of the association between BDNF/CREB signaling by FX extract and antidepressant regulation is needed in future studies. Unfortunately, unlike expected, FXT’s effect on pCREB and BDNF expression was insignificant. In Pinnock et al. (2010), CREB showed higher activity in hippocampus on day 14 than on day 7 of FXT administration (Pinnock et al., 2010). It was concluded that the duration of FXT administration of 10 days was ambiguous to affect the expression or activity of CREB/BDNF.

## Conclusion

In conclusion, reserpine administration induces depressive and anxiety-like behaviors, increasing corticosterone and pro-inflammatory cytokines in the plasma and hippocampus, respectively, and decreasing hippocampal pCREB/BDNF expression. These effects were attenuated by FX extract and its components. Our report suggests that FX leads to anti-inflammatory and neuroprotective effects, including a reduction in pro-inflammatory cytokine concentrations and enhancement of pCREB and BDNF expression in hippocampus, as well as antidepressant and anxiolytic-like effects. Our studies may serve as a preclinical basis that confirm the potential of FX and its components as antidepressants capable of addressing depressive and anxiety-like behaviors caused by monoamine changes. Further studies will be needed to establish mechanisms of action that regulate antidepressant and anxiety-relieving effects.

## Data Availability Statement

All datasets generated for this study are included in the article.

## Ethics Statement

The animal study was reviewed and approved by the Committee on Animal Care of Korea Institute of Oriental Medicine (KIOM 17-104).

## Author Contributions

YRK performed the experiments, analyzed the data, and wrote the manuscript. B-KP performed the experiments and analyzed the data. C-SS performed the HPLC analysis and wrote the manuscript related to HPLC. NSK reviewed the manuscript and discussed reviewer’s comments for manuscript revision. MYL designed the experiments and reviewed the manuscript. All authors have read and approved the manuscript.

## Conflict of Interest

The authors declare that the research was conducted in the absence of any commercial or financial relationships that could be construed as a potential conflict of interest.
